# Whole genome profiling physical map and ancestral annotation of tobacco Hicks Broadleaf

**DOI:** 10.1111/tpj.12247

**Published:** 2013-05-15

**Authors:** Nicolas Sierro, Jan van Oeveren, Michiel J T van Eijk, Florian Martin, Keith E Stormo, Manuel C Peitsch, Nikolai V Ivanov

**Affiliations:** 1 Biological System Research, Philip Morris International R&D, Philip Morris Products SA Quai Jeanrenaud 5, 2000Neuchatel, Switzerland; 2 Keygene NVAgro Business Park 90, 6708 PW, Wageningen, The Netherlands; 3 Amplicon Express Inc.2345 NE Hopkins Court, Pullman, WA, 99163, USA

**Keywords:** physical map, genome, tobacco, *Nicotiana tabacum*, polyploidy, whole-genome profiling, next-generation sequencing

## Abstract

Genomics-based breeding of economically important crops such as banana, coffee, cotton, potato, tobacco and wheat is often hampered by genome size, polyploidy and high repeat content. We adapted sequence-based whole-genome profiling (WGP™) technology to obtain insight into the polyploidy of the model plant *Nicotiana tabacum* (tobacco). *N. tabacum* is assumed to originate from a hybridization event between ancestors of *Nicotiana sylvestris* and *Nicotiana tomentosiformis* approximately 200 000 years ago. This resulted in tobacco having a haploid genome size of 4500 million base pairs, approximately four times larger than the related tomato (*Solanum lycopersicum*) and potato (*Solanum tuberosum*) genomes. In this study, a physical map containing 9750 contigs of bacterial artificial chromosomes (BACs) was constructed. The mean contig size was 462 kbp, and the calculated genome coverage equaled the estimated tobacco genome size. We used a method for determination of the ancestral origin of the genome by annotation of WGP sequence tags. This assignment agreed with the ancestral annotation available from the tobacco genetic map, and may be used to investigate the evolution of homoeologous genome segments after polyploidization. The map generated is an essential scaffold for the tobacco genome. We propose the combination of WGP physical mapping technology and tag profiling of ancestral lines as a generally applicable method to elucidate the ancestral origin of genome segments of polyploid species. The physical mapping of genes and their origins will enable application of biotechnology to polyploid plants aimed at accelerating and increasing the precision of breeding for abiotic and biotic stress resistance.

## Introduction

Tobacco (*Nicotiana tabacum*) is an allotetraploid species (2*n* = 4*x* = 48) and a member of the Solanaceae family, which includes eggplant (*Solanum melongena*), pepper (*Capsicum annuum*), petunia (*Petunia × hybrida*), tomato (*Solanum lycopersicum*) and potato (*Solanum tuberosum*). A tetraploidization event, which occurred approximately 200 000 years ago (Leitch *et al*., 2008) and involved ancestors of *Nicotiana sylvestris* (S genome; 2*n* = 24) and *Nicotiana tomentosiformis* (T genome; 2*n* = 24), is likely to be responsible for its emergence (Lim *et al*., 2004; Clarkson *et al*., 2005). *N. tabacum* therefore has a relatively large genome size (approximately 4500 Mb) compared with other cultivated Solanaceae crops (Arumuganathan *et al*., 1994), and is 50% larger than the human genome.

Analysis of the tobacco genome has been ongoing in the last decade. In 2001, the Tobacco Genome Initiative was established to sequence the tobacco genome using the methyl-filtration method for genome complexity reduction (Rabinowicz *et al*., 1999). The Hicks Broadleaf variety, the breeding background of some flue-cured tobacco cultivars in use today, was chosen as the genotype for generation of bacterial artificial chromosome (BAC) libraries used for sequencing because of its low introgression content. A total of 1 420 169 unique raw Sanger sequences with a mean length of 697 bp were obtained and publicly released (http://www.pngg.org/tgi/index.html). Although the genome assembly of these sequences by the Sol Genomics Network (SGN, http://solgenomics.net/) is very informative, it remains highly fragmented. Its applicability is thus significantly reduced, and a higher-quality draft genome of tobacco is still required.

A high-density tobacco map was built recently by applying simple sequence repeat (SSR) markers generated by the Tobacco Genome Initiative to an F_2_ mapping population derived by crossing the *N. tabacum* Hicks Broadleaf and Red Russian varieties (Bindler *et al*., 2011). This genetic map is comparable in marker density and resolution to the latest tomato and potato genetic maps, and is available from the Sol Genomics Network clade-oriented database containing genomic, genetic, phenotypic and taxonomic information for plants of the Solanaceae and Rubiaceae (coffee) families (Bombarely *et al*., 2011).

Advancements in modern breeding approaches for many economically important crops such as banana (*Musa acuminata*), coffee (*Coffea arabica*), cotton (*Gossypium hirsutum*), potato (*Solanum tuberosum*), tobacco (*Nicotiana tabacum*) and wheat (*Triticum aestivum*) are hampered by genome size, polyploidy and high repeat content. Despite the progress in sequencing technologies achieved in the last decade, assembly of reference polyploid genomes is still challenging. One of the key enabler to achieve this goal is construction of a high-quality physical map (Ariyadasa and Stein, 2012). Recent examples of this approach include cotton and potato. Similar to tobacco, cultivated tetraploid cotton, *Gossypium hirsutum*, arose from combination of the A and D genomes approximately 1–2 million years ago. The whole physical map of *G. hirsutum* has been built using transformation-competent binary BACs and applying the high information content fingerprinting (HICF) method using SNaPshot® (Zhang *et al*., 2012). A physical map of a related cotton species, *Gossypium raimondii*, whose progenitor is the putative contributor of the D genome to cultivated cotton, has also been constructed recently using the HICF method (Lin *et al*., 2010). The *Gossypium* physical maps serve as frameworks for anchoring and ordering the assembled sequences into the reference allotetraploid cotton genome.

Although a reference genome is available for the double-monoploid DM1-3 516 R44 potato (Xu *et al*., 2011), the Potato Genome Sequencing Consortium recognized the necessity of first building a physical map of the heterozygous diploid RH89-039-16 potato using whole genome profiling (WGP™) technology (de Boer *et al*., 2011), before embarking on assembly of a reference genome for the cultivated tetraploid potato. For the large diploid genome of maize (*Zea mays*), construction of a physical map was instrumental in generating chromosome-based pseudomolecules, leading to a first version of the maize B73 reference genome (Wei *et al*., 2009).

To further increase the quality of currently available tobacco resources to that for tomato and potato, we constructed a physical map of tobacco using WGP technology. The constructed physical map is based on sequenced tags of the terminal ends of restriction fragments from pooled BAC clones produced using Illumina's Genome Analyzer II platform and assembled using an adapted FPC program (Soderlund *et al*., 1997; van Oeveren *et al*., 2011). The WGP method significantly differs from the competing SNaPshot® HICF technology (Ding *et al*., 2001; Luo *et al*., 2003, 2010), and delivers a physical map of higher quality and wider utility because of the characteristics and quality of the sequence tags used for physical map assembly (van Oeveren *et al*., 2011). The sequence-based nature of the WGP physical map of tobacco allowed us to determine the ancestral origin of a majority of the tobacco BACs and WGP physical map contigs by comparison with sequence tags obtained from *N. sylvestris* and *N. tomentosiformis*. In addition, we determined the origin of chromosomal regions by linking them to the available tobacco genetic map. Finally, we illustrate the usefulness of the WGP physical map to scaffold DNA sequences by integrating DNA sequences resulting from the Sol Genomics Network assembly of the Tobacco Genome Initiative sequence data.

## Results

### WGP tag generation

A total of 1107 384-well plates from four libraries (425 088 BAC clones) was subjected to WGP™ (Keygene NV, Wageningen, The Netherlands, http://keygene.com), as described by van Oeveren *et al*. (2011). BAC pooling was performed in a two-dimensional format, with each pool consisting of 48 clones. DNA isolated from these pools was digested using *Eco*RI and *Mse*I, bar-coded adapters were ligated, and restriction fragments were amplified. Amplicons were subsequently sequenced from the *Eco*RI restriction site end using the Illumina Genome Analyzer II. In total, this yielded 1718 million reads with a 78 nt read length, a valid sample identification tag and proper restriction site sequence. Deconvolution was performed using the first 31 nt, the first 51 nt and the full 70 nt reads (excluding the bar-code sequences), resulting in half of the total number of reads being assigned as WGP tags to individual BACs. After filtering these WGP tags using several quality criteria, a total of 1.2 million WGP tags were mapped to 361 034 BACs for the 51 nt set (Table 1). The results for all three sets are presented in Table S1. The 51 nt WGP tag analysis was selected as the final dataset because the analysis demonstrated that this read length provided the maximum number of deconvoluted reads.

**Table 1:** Metrics for the physical map construction using a 51 nt tag length

Number of BACs tested	425 088
Genome equivalents	10.4
Number of deconvolutable reads (*M*)	907.7
Number of unique WGP tags	1 239 733
Number of tagged BACs (FPC-ready)	361 034
Percentage of tagged BACs (FPC-ready)	85
Mean number of tags per BAC	32.1
Number of contigs	9750
Number of BACs in contigs	330 632
Number of singletons	30 402
Mean number of BACs per contig	34
N_50_ BACs per contig	60
Mean contig size (Mbp)	0.462
N_50_ contig size (Mbp)	0.689
Genome coverage (Mbp)	4508
Percentage genome coverage	100

### Physical map construction

After conversion of the WGP tag data to pseudo-mobility numbers, the FPC software (Soderlund *et al*., 1997) adapted for use with sequence tags as described by van Oeveren *et al*. (2011) was used to assemble sequence-based physical BAC maps. A cut-off value of 10^−25^ was used with a subsequent DQ step to produce a high-stringency map. Additional end-to-end contig and singleton merging steps at 10^−15^ were performed to obtain a normal-stringency map with fewer contigs. Using the 51 nt set and normal-stringency settings, the tobacco physical map comprised 9750 contigs containing 330 632 BACs (77.8% of the BACs tested; 91.5% of the tagged BACs). Of the remaining BACs, 30 402 did not link to any other BAC and were classified as singletons, and 64 064 BACs had no deconvoluted tags. The estimated mean and N_50_ contig sizes were 462 and 689 kbp, respectively, and the calculated genome coverage was 4508 Mbp (Table 1). The results for both stringency settings for all three sets are presented in Table S2.

### Ancestral origin of tobacco BACs and WGP contigs

**Figure 1:**
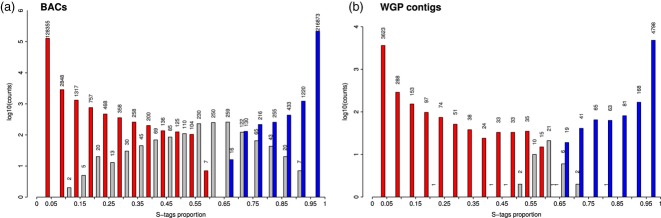
Log_10_ frequency of the weighted proportion of S tags. A value of 0 indicates a T origin (red bars) and a value of one indicates an S origin (blue bars), according to the enrichment *P* values (see Experimental procedures). The gray bars indicate counts of: (a) BACs. (b) WGP contigs of undefined origin, i.e. for which unequivocal assignment to S or T was not possible. Absolute counts are given above each bar.

**Figure 2:**
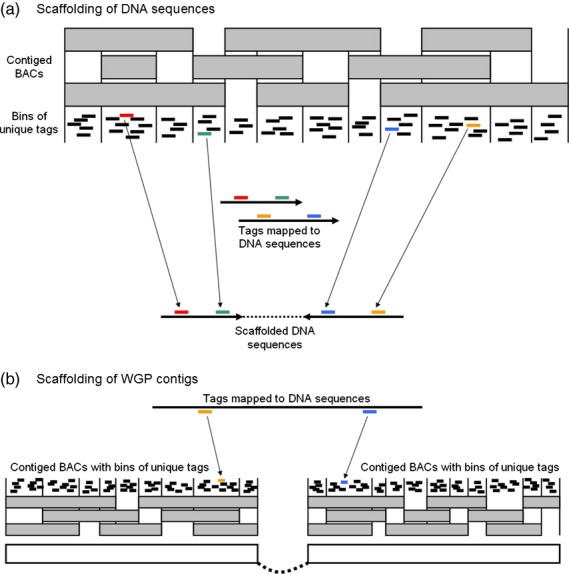
Strategies to scaffold DNA sequences using the WGP physical map and to scaffold WGP contigs using DNA sequences. (a) Scaffolding of DNA sequences using the WGP physical map. The orientation of the scaffolded DNA sequences is indicated by the mapping position of WGP tags from different bins of the same WGP contigs. (b) Scaffolding of WGP contigs using DNA sequences. Tags from end bins of different WGP contigs that map to the same DNA sequence are used to link WGP contigs.

Sequence tags flanking *Eco*RI restriction sites for the whole genomes of *N. sylvestris* and *N. tomentosiformis*, the two closest relatives to the ancestral contributors of the S and T genomes to tobacco, were obtained by digesting genomic DNA of these lines using *Eco*RI and *Mse*I, followed by amplification and Genome Analyzer II sequencing using the same method as applied for WGP map construction of *N. tabacum*. After quality filtering, 1 089 317 *N. sylvestris* and 1 035 343 *N. tomentosiformis* tags were obtained, compared with the 1 239 733 unique 51 nt tags obtained for *N. tabacum* during construction of the physical map, thus allowing determination of the ancestral origin of the tobacco tags, BACs and WGP physical map contigs by *P* value calculations for S or T tag enrichment. This analysis showed that 60.7% of the BACs are of S origin and 37.4% of T origin. The origin of 0.4% of the BACs could not be determined despite their S and/or T tags, and these BACs were classified as having an undefined origin. Finally, 1.5% of the BACs have no S or T tags and are thus of unknown origin. BACs consist of tobacco DNA fragments of approximately 100 kb, and as such should originate from only one of the two ancestors. Being able to assign an ancestral origin to 98.1% of the BACs indicates that S and T tags are not present randomly in BACs, thus validating the origin determination of WGP tags. The *P* value calculations for S or T tag enrichment further showed that 53.7% of the WGP contigs are of S origin, 45.8% are of T origin, 0.5% are of undefined origin, and only four are of unknown origin (Figure 1 and 2). Thus the S and T tags are rarely found together in the same WGP contig, providing additional confidence in the quality of the constructed physical map.

**Table 2:** Ancestral origin of the WGP tags, BACs and contigs

	S origin (%)	T origin (%)	Undefined origin (%)	Unknown origin (%)
WGP tags	494 973 (39.9)	311 399 (25.1)	0 (0.0)	433 361 (35.0)
BACs	219 143 (60.7)	134 933 (37.4)	1375 (0.4)	5583 (1.5)
WGP contigs	5236 (53.7)	4465 (45.8)	45 (0.5)	4 (0.0)

Comparing the ancestral origin of BACs to the ancestral origin of the WGP contigs in which they were placed showed that the majority of the BACs of S or T origin were found in WGP contigs with the same origin annotation (98.0 and 94.0% for BACs of S or T origin, respectively) (Table 3). BACs of undefined origin were almost evenly placed in WGP contigs of S or T origin (43.8 and 53.1%, respectively), whereas 78.1% of the BACs of unknown origin were placed in WGP contigs of T origin and 19.4% in WGP contigs of S origin.

**Table 3:** Number and proportion of BACs classified according to their ancestral origin compared to the ancestral origin of the WGP contigs to which they belong

	WGP contig			
	
BAC	S origin (%)	T origin (%)	Undefined origin (%)	Unknown origin (%)
S origin	199 254 (98.0)	3155 (1.6)	861 (0.4)	0 (0.0)
T origin	6647 (5.3)	117 989 (94.0)	833 (0.7)	0 (0.0)
Undefined origin	491 (43.8)	595 (53.1)	35 (3.1)	0 (0.0)
Unknown origin	150 (19.4)	603 (78.1)	10 (1.3)	9 (1.2)

Taking advantage of the ordering of the BACs in a WGP contig (where no ordering is available for the tags in a BAC) and to complement the enrichment *P* values, the number of domains composed of BACs of S or T origins was estimated for each WGP contig, as described in Experimental procedures. WGP contigs with a clear origin are ideally composed of only one domain, whereas WGP contigs of undefined origin either possess more than one domain or are artifacts created during the WGP physical map construction. The number of domains counted in WGP contigs of S, T or undefined origin showed that only one domain was identified for 94.1 and 93.2% of the WGP contigs of S or T origins, respectively (Table S3). Enrichment *P* values, which are only based on counts and do not consider contig units, are therefore well complemented by the domain structure, as some S and T contigs contain several domains, with the majority corresponding to the assigned origin.

### Linking the genetic and physical maps

The *N. tabacum* genetic map was constructed from a Hicks Broadleaf × Red Russian F_2_ mapping population (Bindler *et al*., 2011). It consisted of 24 linkage groups comprising 1776 unique loci determined by 2318 SSR markers. SSR amplification tests on *N. sylvestris* and *N. tomentosiformis* were then used to annotate tobacco linkage group regions according to their putative S or T ancestral origin. Of the 2318 SSR markers of the *N. tabacum* genetic map, 918 were experimentally linked to 910 BACs. In the WGP physical map, 802 of these BACs were contained in 725 WGP contigs.

The number of BACs and WGP contigs of S, T or undefined origin mapped to these S or T linkage group regions is shown in Table 4. Almost 80 and 65% of the WGP contigs of S or T origins were indicated as linked to S or T linkage group regions, respectively 80% S or 65% T.

**Table 4:** Comparison of determined ancestral origins of BACs and WGP contigs with the putative origin assigned to linkage group regions

	S origin (%)	T origin (%)	Undefined origin (%)	Unknown origin (%)
S linkage group regions				
BACs	331 (78.8)	88 (21.0)	1 (0.2)	0 (0.0)
WGP contigs	308 (79.0)	81 (20.8)	1 (0.3)	0 (0.0)
T linkage group regions				
BACs	118 (30.7)	262 (68.2)	3 (0.8)	1 (0.3)
WGP contigs	128 (35.8)	227 (63.4)	3 (0.8)	0 (0.0)

For each linkage group, Figure S1 shows the location of SSR markers of S or T origin used for the genetic map construction and that of the BACs of S or T origins that are linked to the tobacco genetic map. The linkage groups are colored according to their S or T annotation (based on Bindler *et al*., 2011). For linkage group 22, a color inversion in the S and T assignment is visible in Figure 1 of Bindler *et al*. (2011). Recalculation of ancestral origins after correcting this possible inversion increased the fraction of BACs for which the predicted origin corresponds with the genetic map and decreased the number of conflicting cases by approximately 2% (Table S4).

## Discussion

The WGP physical map of the tobacco genome was constructed from 51 nt WGP tags. Because the Illumina GA sequencing technology that we used produced longer sequence reads, additional maps using longer (70 nt) and shorter (31 nt) tags were also constructed to investigate the influence of tag length on map resolution. Although longer sequence reads may generate more unique WGP tags from the genome, sequence errors over longer read lengths may decrease the actual number of deconvoluted WGP tags based on our criteria of requiring three observations per tag per dimension. Hence, we observed the largest number of deconvoluted reads using 51 nt tags, and chose this tag length as reference for our WGP maps. However, we generated six WGP maps by combining different tag lengths (31, 51 and 70 nt) and FPC stringency levels (‘high’ and ‘normal’ stringency), and concluded that, although small differences in the map metrics were observed, the six maps were highly similar. In general, the use of a higher stringency for physical map construction using FPC resulted in more WGP contigs, obtained in the vast majority of cases by splitting of normal-stringency WGP contigs at points where the BAC coverage is low. Major rearrangements of the BAC orders between the maps were not observed.

Determination of the ancestral origin of WGP tags, BACs and WGP contigs showed that a higher proportion of elements were of S origin than of T origin, regardless of the category. The WGP physical map is expected to cover the whole tobacco genome, and therefore the WGP contigs and the underlying BACs and WGP tags are unlikely to be biased towards one of the two ancestors. The evolutionary distance of *N. sylvestris* and *N. tomentosiformis* from their respective ancestors involved in the hybridization event that gave rise to *N. tabacum* is unclear (Ren and Timko, 2001; Moon *et al*., 2008). Assuming that after hybridization both ancestral genomes evolved at the same rate in tobacco, the proportions of WGP tags of S or T origin indicate that *N. sylvestris* is closer to the S ancestor than *N. tomentosiformis* is to the T ancestor. The relationship between *N. tabacum* and *N. sylvestris* is well established, whereas that between *N. tabacum* and *N. tomentosiformis* has been challenged frequently by proposals of a third ancestral *Nicotiana* species, *N. otophora* (Ren and Timko, 2001; Murad *et al*., 2002). Extending the current work (i.e. tag sequencing based on genomic DNA) to the latter species may help to resolve this issue.

An initial conclusion that may be drawn from Table 2 is that one-third of the *N. tabacum* (Hicks Broadleaf) WGP tags are not present or are undetectable in *N. sylvestris* or *N. tomentosiformis*. At the BAC level, it was shown that the proportion of BACs for which no origin could be determined was 1.5%. The presence of 0.4% of BACs with an undefined origin is not surprising, and this small fraction is hypothesized to correspond largely to BACs covering regions where recombination between the S and T ancestral genomes has occurred. At the physical map level, 0.5% of the contigs have an undefined origin. Although a proportion of this fraction may represent contigs that were constructed inappropriately, this is expected to occur because the mean size of the region covered by a contig is larger than that covered by a BAC, and hence there are more chances that it covers a cross-over point.

Although not in full agreement, the ancestry annotation of the SSR markers used to construct the genetic map of tobacco (Bindler *et al*., 2011) and the predicted origin of the BACs linked to these markers correspond to a large extent. This indicates that the tag-based approach is appropriate to infer the ancestral origin of a BAC or a WGP contig.

In addition to the tobacco WGP map presented here, WGP physical maps of two other Solanaceae crops, potato and tomato, have been constructed (de Boer *et al*., 2011; Orozco López, 2012). Table S5 summarizes the metrics for the three maps. To accommodate its larger genome size, longer tags were used for tobacco than for tomato and potato. Because of the differences in genome sizes, it is better to compare these numbers relative to total genome coverage. In this case, the number of unique tags per Mb is similar, at 274.8, 286.6 and 275.0 for tomato, potato and tobacco, respectively, whereas the number of tagged BACs is larger for tobacco (80.1) than for tomato (69.3) and potato (59.2). After construction of the physical maps, 2.2 contigs per Mb were obtained for tobacco, which is less than the 2.6 and 3.1 contigs obtained for tomato and potato, respectively. Similarly, the number of singletons per Mb for tobacco (6.7) is less than that for tomato (14.1) and potato (7.7). The mean and N_50_ contig sizes, both in terms of BACs and kbp, are also higher for the tobacco map than for the tomato and potato maps. Collectively, these values indicate that the WGP physical map of tobacco is of comparable, or even slightly superior, quality compared with the tomato and potato physical maps.

The sequence-based WGP contigs may be used to scaffold DNA sequences, and, via a genetic map, to assign them to pseudo-chromosomes. At the same time, DNA sequences may assist in scaffolding of WGP contigs. A schematic illustration of how WGP contigs may be used to scaffold DNA sequences by taking advantage of the sequenced WGP tags is shown in Figure 2(a). We applied this strategy to the Sol Genomics Network assembly of the publicly available Tobacco Genome Initiative reads to confirm its feasibility, and obtained 167 scaffolds of two contigs, 23 scaffolds of three contigs, nine scaffolds of four contigs, and one scaffold of five contigs. The Sol Genomics Network assembly has a mean contig size of 1275 bp, which is shorter than the estimated mean distance between two WGP tags (approximately 3 kb). Because two mapped WGP tags from different regions of one WGP contig are necessary to obtain orientation information for a sequence in a scaffold, a large proportion of the Sol Genomics Network assembly contigs are probably not usable because of their short size. The inability to order tags within a WGP contig region limits the efficiency of the scaffolding in two ways. First, it prevents an accurate estimation of the distance between the scaffolded contigs, giving an approximate range only. Second, it prohibits scaffolding of contigs that are mapped to the same regions, effectively making scaffolding using WGP contigs a tool for long-range scaffold construction. As such, WGP in its current configuration is complementary to other DNA sequence scaffolding approaches that target shorter scaffolding distances, such as EST- and mate pair library-based scaffolding. Adaptations of the WGP protocol, e.g. use of other restriction enzymes that cut more frequently and thus increase the tag density, may enhance these features of WGP. The BAC coverage and its variation along the WGP contig may be used to assess the confidence that two contigs should be scaffolded, where low coverage indicates possible mis-assembly of the WGP contig.

Although perhaps of less direct interest, WGP contigs and/or singleton BACs themselves may be scaffolded using DNA sequences. Such a strategy is illustrated in Figure 2(b). Its use to scaffold contigs from the WGP physical map using contigs from the Sol Genomics Network assembly of the publicly available Tobacco Genome Initiative reads resulted in 17 scaffolds of two WGP contigs. As mentioned previously, the Sol Genomics Network assembly has a mean contig size of only 1275 bp, and, at 37 736 bp, its longest contig covers only approximately one-third of a BAC. Again, the abundance of small contigs reduces the possibilities of scaffolding. Nevertheless, the few scaffolds obtained are sufficient to demonstrate how DNA sequences may be used to scaffold WGP contigs. High-quality scaffolding will of course require longer DNA sequences and more stringent criteria to avoid erroneous scaffolding. Using multiple tags from different bins for each scaffolded WGP contig would be a first step in that direction.

A high-quality physical map of tobacco was constructed using WGP with metrics equivalent to those of tomato and potato. Benefitting from the fact that this map is based on sequenced DNA tags, and despite the lack of a full genome sequence, the ancestral origin of the BACs used in the physical map and the obtained WGP contigs was determined in 98 and 99% of the cases, respectively. When linked to the tobacco genetic map, this ancestry assignment was found to be in agreement with the results obtained with SSR markers. As is the case for other plants, the tobacco physical map may be used for DNA sequence scaffolding, and will thus be of use in *de novo* sequencing and assembly of the tobacco genome. The combination of WGP map construction and tag sequencing of (putative) ancestral lines may be beneficial to unravel ancestry relationships in other polyploid organisms, and does not require prior sequence information to obtain high-resolution ancestry information.

Agricultural crops grown in the field are frequently prone to abiotic stresses (Tester and Bacic, 2005; Vinocur and Altman, 2005; Mittler, 2006; Vij and Tyagi, 2007) and biotic stresses (Peterson and Higley, 2000; Tolmay, 2001; Cattivelli *et al*., 2008; Reynolds and Tuberosa, 2008), which affect the yield, quality, harvesting time and other valuable characteristics. Genes conferring resistance to these stresses found in related wild plant species may be transferred to cultivars by utilizing numerous molecular biology methods. Physical mapping of such genes in naturally occurring *Nicotiana* species with the help of the tobacco physical map will facilitate plant biotechnology applications aimed at accelerating and increasing the precision of plant breeding for abiotic and biotic stress resistance.

## Experimental procedures

### BAC libraries

Four BAC libraries of the allotetraploid genome of *N. tabacum* cv. Hicks Broadleaf (inbred line PI 552397 or TC 311 in the United States Department of Agriculture, Agricultural Research Service, Germplasm Resources Information Network (USDA-ARS-GRIN database, http://www.ars-grin.gov/), comprising a total of 425 088 BAC clones (1107 384-well plates) and approximately 10.4-fold genome coverage, were used for construction of the WGP map. These libraries were (i) a *Hin*dIII library (Microsynth AG, Balgach, Switzerland, http://www.microsynth.ch) consisting of 112 896 clones with an estimated mean insert size of 100 kb, representing approximately 2.5-fold genome coverage, (ii) a *Bam*HI library (Microsynth), consisting of 146 304 clones with an estimated mean insert size of 100 kb, representing approximately 3.4-fold genome coverage, (iii) an *Eco*RI library consisting of 69 120 clones, with an estimated mean insert size of 125 kb, representing approximately 1.9-fold genome coverage, and (iv) a *Hin*dIII library, consisting of 96 768 clones with an estimated mean insert size of 125 kb, representing approximately 2.7-fold genome coverage.

### BAC library pooling, DNA isolation and WGP sample preparation

Individual BACs of the four libraries stored in 384-well plates were pooled in a 2D format prior to DNA isolation. Specifically, BACs were 2D-pooled by taking six plates at a time in a 2 × 3 layout (termed a superpool) and pooling each row over two plates (48 BACs) and each column over three plates (48 BACs), yielding 96 pools per superpool. BAC pools were subjected to isolation of high-concentration DNA, and WGP sample preparation was performed essentially as described by van Oeveren *et al*. (2011). Briefly, AFLP® templates (Vos *et al*., 1995) were prepared from pooled BAC DNA by digestion using five units of *Eco*RI and two units of *Mse*I. Next, adapter ligation was performed using either a P5 *Eco*RI adapter containing a 5 or 6 nt sample identification tag in combination with a unique P7 *Mse*I adaptor (when one or two superpools were pooled per lane for sequencing), or using a P5 *Eco*RI adaptor containing a 5 nt sample identification tag and P7 *Mse*I adaptor with a 3 nt sample identification tag (when three superpools were pooled for sequencing in one lane). PCR was performed in 20 μl volumes, and contained 5 μl of 10-fold diluted restriction ligation mixture, 30 ng Illumina P5 primer (5′-AATGATACGGCGACCACCG-3′), 30 ng Illumina P7 primer (5′-CAAGCAGAAGACGGCATACGA-3′), 0.2 mm dNTPs, 0.4 units of Amplitaq (Applied Biosystems, http://www.appliedbiosystems.com) and 1× Amplitaq buffer. Next, equal amounts of BAC pool PCR reaction mixtures were pooled per superpool, and purified using a QIAquick PCR purification kit (Qiagen, http://www.qiagen.com). When one or two superpools were sequenced per lane, each BAC pool was bar-coded using one of 192 different 5 or 6 nt sample identification tags in the P5 *Eco*RI adapter. When three different superpools were combined for sequencing in one lane, each BAC pool was tagged by a combination of one of 96 different P5 *Eco*RI adapters with a 5 nt sample identification tag, and one of three different P7 *Mse*I adapters with a 3 nt sample identification tag, such that 288 BAC pools could be pooled per lane for sequencing.

### Sequencing

A total of 90 lanes of either 76 or 78 cycles of sequencing, divided over 15 runs, were performed using an Illumina (San Diego, CA, USA, http://www.illumina.com) Genome Analyzer II. Sequencing with 76 cycles was used when one or two superpools were pooled per lane, whereas 78-cycle sequencing with a second priming event at the *Mse*I side was used when three superpools were pooled per lane, in which case the 3 nt sample identification tags at the *Mse*I side were determined. Each run used a flow cell with eight lanes of physically separated samples, with the same set of sample tags used for each lane. Genome Analyzer II runs were performed with seven lanes of tobacco WGP samples at 5.5 pm concentration, each covering one, two or three superpools represented in 96, 192 or 288 row and column pools, respectively. The Illumina pipeline software was used to extract sequence reads of 76 or 78 nt length from the images. An additional quality filter was applied to select only those reads for which all base calls have at least a Solexa quality of 0 (equivalent to a Phred quality of 3, http://www.phrap.com/phred) on the Illumina GA scale. All sequence data have been deposited in the European Nucleotide Archive (ENA) under accession number ERP001765.

### Deconvolution and filtering

Sequence reads were split into three parts in the case of 76-cycle sequencing or in four parts in the case of 78-cycle sequencing to enable assignment of unique tags to pools and to allow consecutive deconvolution into individual BACs. In the case of 76-cycle sequencing, the first 5 or 6 nt represented the sample (i.e. BAC pool) identification tag at the *Eco*RI side, the next 6 nt matched the *Eco*RI restriction site of the adapter, and the remaining nucleotides defined the ‘WGP tag’. In the case of 78-cycle sequencing with combinatorial bar-coding at the *Eco*RI and *Mse*I side ends, a fourth section representing the 3 nt sample identification tag at the *Mse*I side was obtained.

Three different WGP tag lengths were analyzed to test the effect of read length on WGP map resolution: 31, 51 and 70 nt (all including the *Eco*RI restriction site). The assignment of unique WGP tags to individual BACs was based on the following criteria. First, a specific WGP tag must occur in two pools to indicate its unique position on the plate – one column and one row pool with both being represented by at least three reads. Second, if WGP tags are inadvertently observed in a third or fourth pool, the number of reads in these other pools must be less than a tenth of those in the smallest true pool. WGP tags not matching these criteria were discarded. A computer script was used to recognize and trim the sample identification tags and the restriction site part of the sequence reads and to perform the deconvolution. Unique WGP tags were defined by grouping them in 100% identical read sets. The output of this procedure consisted of a list of all WGP tags, the corresponding number of reads, and the identification number of the BAC they were assigned to. Finally, a filtering step was applied to remove WGP tags matching vector, *Escherichia coli* or chloroplast sequences, those containing homopolymer sequences of 5 nt or longer, and WGP tags occurring on just a single BAC.

### Contig building

Contig building was performed using the FPC program (Soderlund *et al*., 1997). This software tool was originally developed for analyzing BAC fingerprint data, i.e. restriction fragments determined by their length. The WGP tags were adapted for use in FPC as described by van Oeveren *et al*. (2011) by converting them into numbers to yield pseudo restriction fragment sizes for which the software was originally designed. As the WGP tags are uniquely defined by their sequence composition, FPC was used at the highest stringency setting of tolerance (value = 0). Various cut-off values were tested, specifying the threshold on the probability of BAC coincidence, i.e. the likelihood that different BACs containing partly overlapping sets of WGP tags originate from the same genomic region. The output of FPC consisted of a list with contigs and the corresponding order of BACs within each contig. The genome coverage, mean contig size and N_50_ contig size in million base pairs were estimated by multiplying FPC band units by the mean distance between two WGP tags. The latter was estimated by dividing the mean BAC insert size by the mean number of WGP tags.

### Linking of BACs to the genetic map

To identify BACs containing SSR markers, 1185 SSR primer pairs were screened from pools and superpools of 196 *Bam*HI and 193 *Hind*III BAC library plates representing approximately threefold coverage of the tobacco genome (approximately 149 000 BACs).

### Determination of ancestral origin

Nuclear genomic DNA was isolated from seedling leaf material of *N. sylvestris* TW138 and *N. tomentosiformis* TW142 in accordance with a protocol for high-molecular-weight nuclear DNA extraction (Liu and Whittier, 1994). As the *N. tomentosiformis* line did not yield enough nuclear DNA, genomic DNA isolation was performed using the Nucleon plant genomic DNA extraction kit (Hologic Gen-Probe Inc., Bedford, MA, USA, http://www.gen-probe.com) according to the manufacturer's instructions to obtain sufficient material. DNA samples were used for *Eco*RI/*Mse*I (E/M) AFLP template preparation as described by Vos *et al*. (1995), using two different bar-coded adaptors suitable for Genome Analyzer II sequencing, in a similar approach to the WGP sample preparation. Seven lanes of a single Genome Analyzer II run were used with a read length of 75 nt, yielding 63.9 million reads for these two samples. Reads for which one or more sequenced nucleotide had a phred quality score lower than 20 (i.e. a probability of error of 0.01) were discarded. For both ancestral species, unique sequences of 51 nt length were grouped into tags, and tags found in both species or with only one read were removed.

The S or T ancestral origin of each BAC included in the physical map construction and each WGP contig was determined by calculating the S or T enrichment *P* values. The proportion of S tags and T tags, weighted according to the number of reads in *N. sylvestris* and *N. tomentosiformis*, respectively, was computed for each BAC or WGP contig. If the BAC or WGP contig was enriched with S or T tags), the estimated proportion was far from a random selection of tags from the original pool. Therefore, an enrichment *P* value for the S and T tag proportions was estimated using the hypergeometric distribution for the null hypothesis. The *P* values obtained were subsequently corrected for multiple testing effects using the Benjamini–Hochberg false discovery rate (Benjamini and Hochberg, 1995). The ancestral origin of a BAC or WGP contig was predicted to be S or T if the enrichment *P* value for S or T was <10^−6^, and the enrichment *P* value for T or S, respectively, was above 10^−6^, otherwise the origin was undefined.

To elucidate the ancestral structure of a WGP contig, each WGP contig unit, as defined by the FPC software, was assigned a putative label S or T if the number of S or T BACs covering the unit exceeds the 95% quantile of a binomial distribution with parameter 0.5. Otherwise the unit was labeled ‘undefined’. The advantage of using this quantile over a constant threshold is that it penalizes low-coverage regions of the WGP contig.

Once putative labels were assigned, the WGP contig was split into connected S, T or undefined segments by mimicking a classification and regression tree approach (e.g. Hastie *et al*., 2001). To control the level of smoothing, a minimum segment size of 40 was chosen. The binary rules obtained were then parsed to extract the structure of the WGP contig. Each segment of a given origin was called a domain of the WGP contig.

### Scaffolding

The position on a WGP contig covered by all the BACs containing a given WGP tag, and by no other BACs, was calculated for all WGP tags belonging to only one WGP contig. These WGP tags were mapped to the Sol Genomics Network version 1 assembly of the Tobacco Genome Initiative reads, and perfect matches were retained. Assembly contigs with at least two mapped WGP tags from two parts of the same WGP contig were selected, as they could be mapped with orientation to the WGP contig. Non-overlapping assembly contigs mapping to the same WGP contig were scaffolded. Assembly contigs with mapped WGP tags from two different WGP contigs were used to scaffold these WGP contigs. The positions of the WGP tags and of the BACs in the 51 nt normal-stringency WGP physical map are shown in Tables S6 and S7, respectively.

## SUPPORTING INFORMATION

**Figure S1.** SSR and BAC markers of S or T origin used for the genetic map construction.

**Table S1.** Number of BACs and WGP tags for the physical map construction using 31, 51 or 70 nt tags.

**Table S2.** Metrics of the normal- and high-stringency physical maps constructed using 31, 51 or 70 nt tags.

**Table S3.** Distribution of domains of S or T genome origin counted in WGP contigs of S, T or undefined origin.

**Table S4.** Comparison of determined ancestral origins of BACs and WGP contigs to the putative origin assigned to linkage group regions following correction for the inversion of the S and T annotation of linkage group 22.

**Table S5.** Metrics for the WGP physical maps of tomato, potato and tobacco.

**Table S6.** Positions of the BACs in the 51 nt normal-stringency WGP physical map of the tobacco genome.

**Table S7.** Position and ancestral origin of WGP tags in the 51 nt normal-stringency WGP physical map of the tobacco genome.
