# Supplementary material for: Whole genome profiling physical map and ancestral annotation of tobacco Hicks Broadleaf
Source: Plant J. 2013 May 15;75(5):880–9. doi: 10.1111/tpj.12247 (PMC3824204; doi:10.1111/tpj.12247)
Supplement: Supplementary file 1 [file tpj0075-0880-SD1.docx]

**Legends for the Supplementary Material**

**Figure S1.** SSR (left) and BAC (right) markers of S or T origin used for the genetic map construction. Linkage groups are colored according to their S or T annotation from Bindler et al. (6)

**Table S1**. Number of BACs and WGP tags for the physical map construction using 31, 51 or 70 nt tags

**Table S2**. Metrics of the normal and high stringency physical maps constructed with 31, 51 or 70 nt tags

**Table S3**. Distribution of domains of S or T genome origin counted in WGP contigs of S, T or undefined origin. The maximum number of domains is six, composed of undefined and a single ancestor (S or T)

**Table S4**. Comparison of determined ancestral origins of BACs and WGP contigs to the putative origin assigned to linkage group regions following correction for the inversion of the S and T annotation of linkage group 22

**Table S5**. Metrics for the WGP physical maps of tomato, potato and tobacco

**Table S6.** Positions of the BAC in the 51nt normal stringency WGP physical map of the tobacco genome. Columns are the BAC number, the WGP contig, the start position in the WGP contig, and the end position in the WGP contig.

**Table S7.** Position and ancestral origin of WGP tag in the 51nt normal stringency WGP physical map of the tobacco genome. Columns are the WGP tag number, the ancestral origin of the WGP tag, the WGP contig, the start position in the WGP contig, and the end position in the WGP contig.
