# Supplementary material for: Whole genome profiling physical map and ancestral annotation of tobacco Hicks Broadleaf
Source: Plant J. 2013 May 15;75(5):880–9. doi: 10.1111/tpj.12247 (PMC3824204; doi:10.1111/tpj.12247)
Supplement: Supplementary file 2 [file tpj0075-0880-SD2.docx]

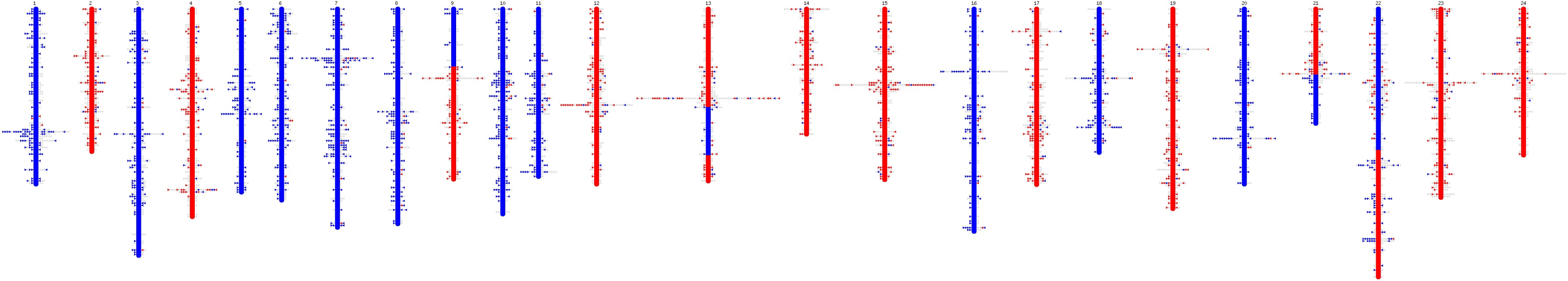


**Fig. S1.** SSR (left) and BAC (right) markers of S or T origin used for the genetic map construction. Linkage groups are colored according to their S or T annotation from Bindler et al. (6)
