# Supplementary material for: Whole genome profiling physical map and ancestral annotation of tobacco Hicks Broadleaf
Source: Plant J. 2013 May 15;75(5):880–9. doi: 10.1111/tpj.12247 (PMC3824204; doi:10.1111/tpj.12247)
Supplement: Supplementary file 3 [file tpj0075-0880-SD3.docx]

**Table S1**. Number of BACs and WGP tags for the physical map construction using 31, 51 or 70 nt tags

| Tag length (nt) | 31 | 51 | 70 |
| --- | --- | --- | --- |
| Number of BACs tested | 425,088 | 425,088 | 425,088 |
| Genome equivalents | 10.4 | 10.4 | 10.4 |
| Number of deconvolutable reads (M) | 799.5 | 907.7 | 871.1 |
| Number of unique WGP tags | 1,077,412 | 1,239,733 | 1,264,201 |
| Number of tagged BACs (FPC ready) | 359,390 | 361,034 | 363,196 |
| Tagged BACs (FPC ready) (%) | 85 | 85 | 85 |
| Average number of tags/BAC | 30.3 | 32.1 | 30.5 |
