# Supplementary material for: Whole genome profiling physical map and ancestral annotation of tobacco Hicks Broadleaf
Source: Plant J. 2013 May 15;75(5):880–9. doi: 10.1111/tpj.12247 (PMC3824204; doi:10.1111/tpj.12247)
Supplement: Supplementary file 4 [file tpj0075-0880-SD4.docx]

**Table S2**. Metrics of the normal and high stringency physical maps constructed with 31, 51 or 70 nt tags

| Tag length (nt) | 31 | 31 | 51 | 51 | 70 | 70 |
| --- | --- | --- | --- | --- | --- | --- |
| Stringency | normal | high | normal | high | normal | high |
| Number of contigs | 10,994 | 12,720 | 9,750 | 11,390 | 10,515 | 12,212 |
| Number of BACs in contigs | 326,901 | 293,220 | 330,632 | 299,187 | 328,583 | 295,529 |
| Number of singletons | 32,489 | 66,170 | 30,402 | 61,847 | 34,613 | 67,667 |
| Average no. of BACs/contig | 30 | 23 | 34 | 26 | 31 | 24 |
| N_50_ BACs/contig | 51 | 41 | 60 | 47 | 55 | 43 |
| Average contig size (Mbp) | 0.426 | 0.375 | 0.462 | 0.403 | 0.447 | 0.392 |
| N_50_ contig size (Mbp) | 0.614 | 0.535 | 0.689 | 0.588 | 0.659 | 0.566 |
| Genome coverage (Mbp) | 4,682 | 4,776 | 4,508 | 4,592 | 4,700 | 4,792 |
| Genome coverage (%) | 104 | 106 | 100 | 102 | 104 | 106 |
