# Supplementary material for: Whole genome profiling physical map and ancestral annotation of tobacco Hicks Broadleaf
Source: Plant J. 2013 May 15;75(5):880–9. doi: 10.1111/tpj.12247 (PMC3824204; doi:10.1111/tpj.12247)
Supplement: Supplementary file 5 [file tpj0075-0880-SD5.docx]

**Table S3**. Distribution of domains of S or T genome origin counted in WGP contigs of S, T or undefined origin. The maximum number of domains is six, composed of undefined and a single ancestor (S or T)

|  | WGP contig | | |  |
| --- | --- | --- | --- | --- |
| Domain structure | S origin | T origin | Undefined origin | Unknown origin |
| 1-S | 4925 (94.1%) | 1 (0.0%) | 1 (2.2%) | 0 (0.0%) |
| 1-T | 0 (0.0%) | 4160 (93.2%) | 0 (0.0%) | 0 (0.0%) |
| 1-undefined | 0 (0.0%) | 2 (0.0%) | 7 (15.6%) | 4 (100.0%) |
| 2-S/T | 73 (1.4%) | 30 (0.7%) | 10 (22.2%) | 0 (0.0%) |
| 2-S/undefined | 17 (0.3%) | 0 (0.0%) | 1 (2.2%) | 0 (0.0%) |
| 2-T/S | 59 (1.1%) | 21 (0.5%) | 4 (8.9%) | 0 (0.0%) |
| 2-T/undefined | 1 (0.0%) | 39 (0.9%) | 4 (8.9%) | 0 (0.0%) |
| 2-undefined/S | 31 (0.6%) | 0 (0.0%) | 0 (0.0%) | 0 (0.0%) |
| 2-undefined/T | 0 (0.0%) | 69 (1.5%) | 1 (2.2%) | 0 (0.0%) |
| 3-S/T/S | 8 (0.2%) | 0 (0.0%) | 0 (0.0%) | 0 (0.0%) |
| 3-S/T/undefined | 1 (0.0%) | 0 (0.0%) | 0 (0.0%) | 0 (0.0%) |
| 3-S/undefined/S | 38 (0.7%) | 1 (0.0%) | 1 (2.2%) | 0 (0.0%) |
| 3-S/undefined/T | 35 (0.7%) | 22 (0.5%) | 6 (13.3%) | 0 (0.0%) |
| 3-T/S/T | 2 (0.0%) | 9 (0.2%) | 0 (0.0%) | 0 (0.0%) |
| 3-T/S/undefined | 1 (0.0%) | 0 (0.0%) | 0 (0.0%) | 0 (0.0%) |
| 3-T/undefined/S | 33 (0.6%) | 15 (0.3%) | 5 (11.1%) | 0 (0.0%) |
| 3-T/undefined/T | 1 (0.0%) | 67 (1.5%) | 1 (2.2%) | 0 (0.0%) |
| 3-undefined/S/T | 0 (0.0%) | 2 (0.0%) | 0 (0.0%) | 0 (0.0%) |
| 3-undefined/S/undefined | 2 (0.0%) | 0 (0.0%) | 0 (0.0%) | 0 (0.0%) |
| 3-undefined/T/S | 0 (0.0%) | 1 (0.0%) | 0 (0.0%) | 0 (0.0%) |
| 3-undefined/T/undefined | 0 (0.0%) | 9 (0.2%) | 1 (2.2%) | 0 (0.0%) |
| more than 3 | 9 (0.2%) | 17 (0.4%) | 3 (6.7%) | 0 (0.0%) |
