# Supplementary material for: Whole genome profiling physical map and ancestral annotation of tobacco Hicks Broadleaf
Source: Plant J. 2013 May 15;75(5):880–9. doi: 10.1111/tpj.12247 (PMC3824204; doi:10.1111/tpj.12247)
Supplement: Supplementary file 6 [file tpj0075-0880-SD6.docx]

**Table S4**. Comparison of determined ancestral origins of BACs and WGP contigs to the putative origin assigned to linkage group regions following correction for the inversion of the S and T annotation of linkage group 22

|  | S origin | T origin | Undefined origin | Unknown origin |
| --- | --- | --- | --- | --- |
| S linkage group regions | | | | |
| BACs | 342 (80.5%) | 81 (19.1%) | 2 (0.5%) | 0 (0.0%) |
| WGP contigs | 316 (80.4%) | 76 (19.3%) | 1 (0.3%) | 0 (0.0%) |
| T linkage group regions | | | | |
| BACs | 107 (28.2%) | 269 (71.0%) | 2 (0.5%) | 1 (0.3%) |
| WGP contigs | 119 (33.5%) | 233 (65.6%) | 3 (0.8%) | 0 (0.0%) |
