# Supplementary material for: Whole genome profiling physical map and ancestral annotation of tobacco Hicks Broadleaf
Source: Plant J. 2013 May 15;75(5):880–9. doi: 10.1111/tpj.12247 (PMC3824204; doi:10.1111/tpj.12247)
Supplement: Supplementary file 7 [file tpj0075-0880-SD7.docx]

**Table S5**. Metrics for the WGP physical maps of tomato, potato and tobacco

|  | Tomato | Potato | Tobacco |
| --- | --- | --- | --- |
| Genome size (Mbp) | 950 | 840 | 4,500 |
| Genome equivalents BACs tested | 10.9 | 11.4 | 10.4 |
| Tag length (nt) | 26 | 26 | 51 |
| FPC ready unique tags (per Mbp covered) | 261,913 (274.8) | 322,437 (286.6) | 1,239,733 (275.0) |
| FPC ready tagged BACs (per Mbp covered) | 66,084 (69.3) | 66,545 (59.2) | 361,034 (80.1) |
| WGP tags per BAC | 33 | 34 | 32 |
| Contigs (per Mbp covered) | 2,521 (2.6) | 3,459 (3.1) | 9,750 (2.2) |
| BACs in contig (per Mbp covered) | 52,617 (55.2) | 57,890 (51.5) | 330,632 (73.3) |
| Singletons (per Mbp covered) | 13,467 (14.1) | 8,655 (7.7) | 30,402 (6.7) |
| Average contig size (BACs) | 21 | 17 | 34 |
| N_50_ BACs per contig | 26 | 30 | 60 |
| Average contig size (kbp) | 378 | 325 | 462 |
| N_50_ contig size (kbp) | 563 | 465 | 689 |
| Total genome coverage (Mbp and % of genome) | 953 (100%) | 1,125 (134%) | 4,508 (100%) |
